# Supplementary figures and images for: Defined roles for the Staphylococcus aureus POT transporter DtpT in di/tripeptide uptake and glutathione utilisation inside human macrophages
Source: PLoS Pathog. 2025 Sep 26;21(9):e1013535. doi: 10.1371/journal.ppat.1013535 (PMC12510641; doi:10.1371/journal.ppat.1013535)

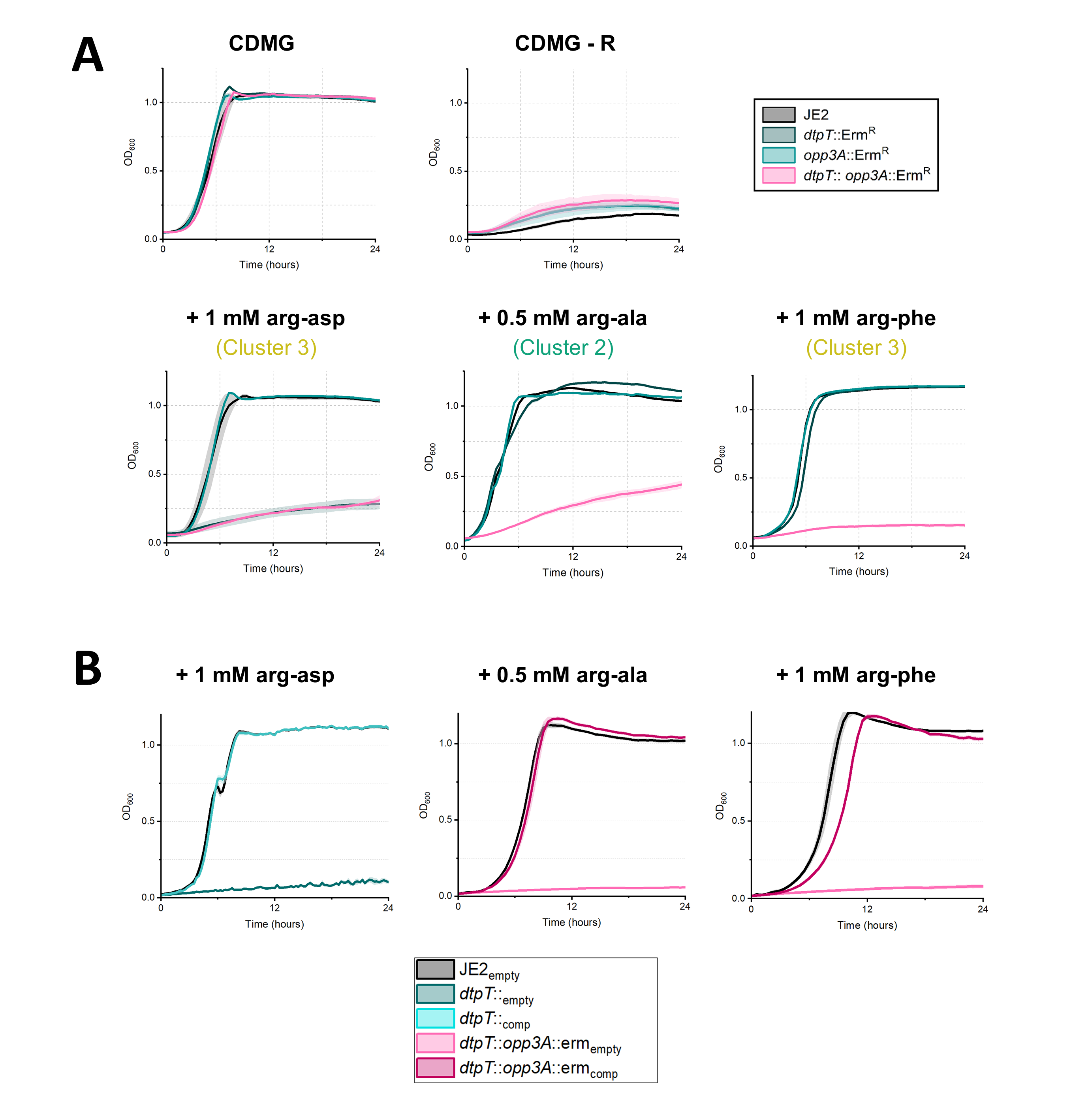

Supplement: S1 Fig — (A) Growth of S. aureus strain JE2 and mutant derivatives in CDMG, CDMG – R and CDMG – R supplemented with three dipeptides, as labelled. Corresponding PM clusters are also provided. (B) Growth of deficient strains is restored by in-trans expression of dtpT under its native promoter in each case. In each case, strains were grown over 24 hours and OD600 was measured every 30 minutes for three biological replicates. Curves indicate the mean values for each reading and the standard deviation in each case is indicated by the shaded area. (TIF) [file ppat.1013535.s001.tif]

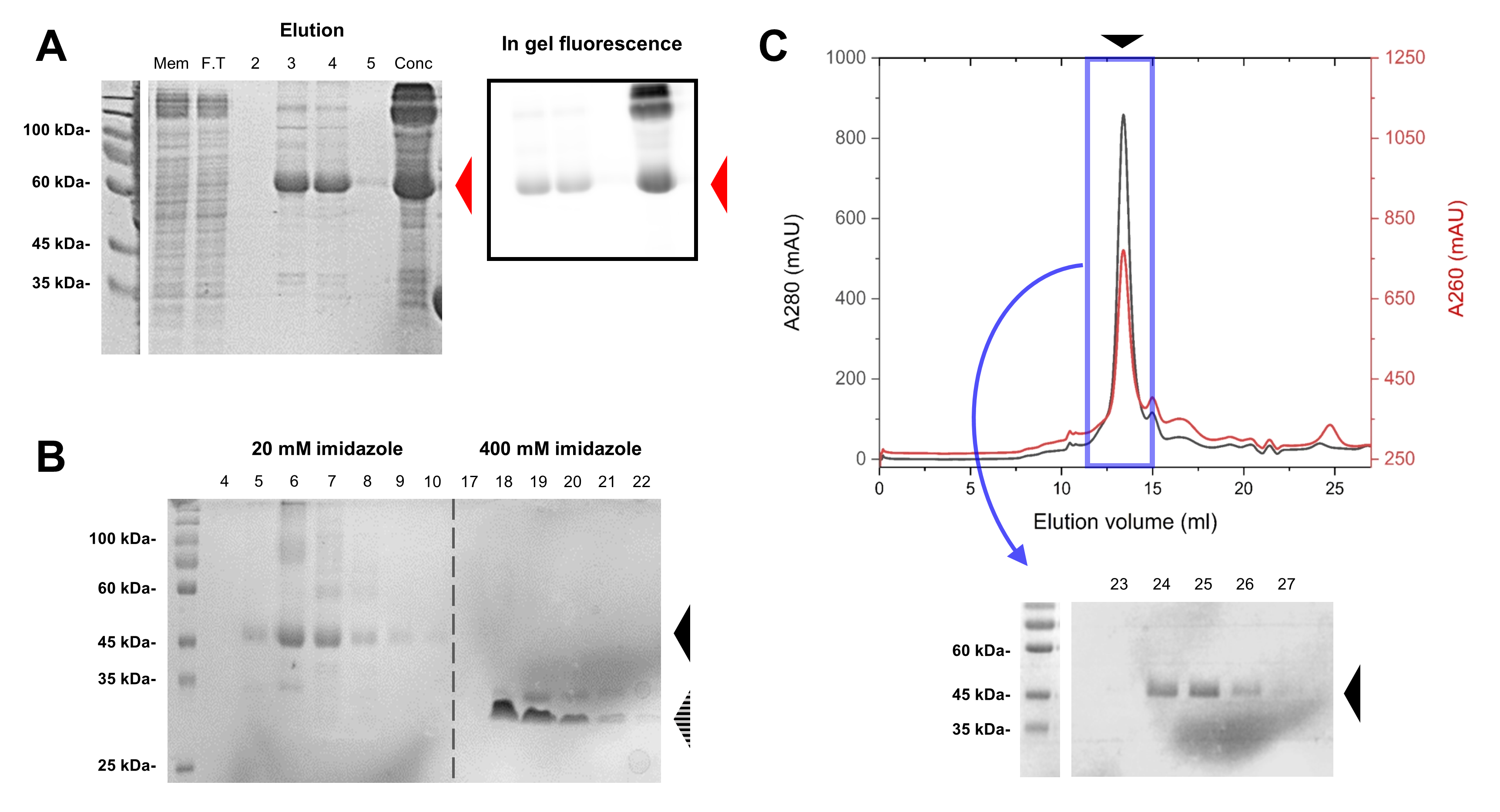

Supplement: S2 Fig — (A) Nickel affinity purification of a DtpT-GFP fusion protein. Images correspond to total protein (left; Coomassie blue stain) and DtpT-GFP only (right; in-gel GFP fluorescence). Mem = Membrane fraction. F.T = Column flow-through. Conc = concentrated fractions. (B) Representative gel image of a reverse nickel affinity purification of untagged DtpT after TEV cleavage of GFP-8His. (C) SEC purification of pure DtpT protein following TEV cleavage. A single sharp peak is seen at approximately 12.5 ml elution volume in the A280 trace (upper) corresponding to the pure DtpT protein, split across elution fractions 24–26 (lower). Overall, approximately 2.35 mg of pure DtpT protein was yielded from 2 L of bacterial culture as estimated from A280. The black arrow in each case indicates the position of a band corresponding to the pure DtpT protein. The red arrow indicates the position of a band corresponding to the expected DtpT-GFP fusion protein. The striped arrow indicates the position of sfGFP. (TIF) [file ppat.1013535.s002.tif]

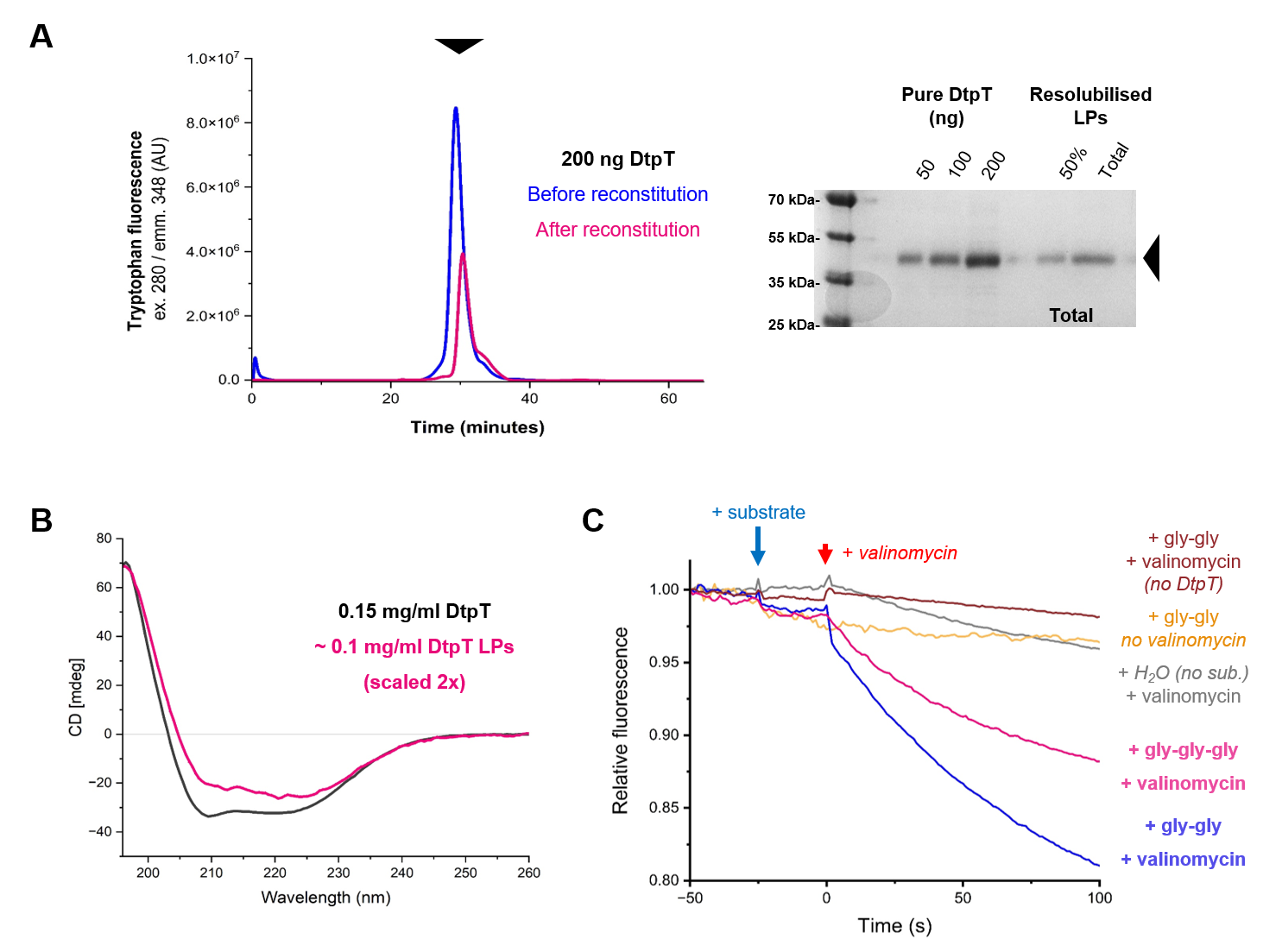

Supplement: S3 Fig — (A) Successful reconstitution of DtpT was confirmed by size exclusion chromatography (left) and SDS PAGE (right). The expected position of the soluble DtpT protein is indicated by the black arrow. (B) CD spectra of pure DtpT (grey) and DtpT liposomes (pink). A scaling factor of 2x has been applied to the DtpT-liposome spectrum to facilitate comparison with the detergent-soluble protein. (C) Validation of pyranine assays in DtpT liposomes. Curves show the mean of three replicates normalised to the initial fluorescence signal (t = -50 s). In each case, peptide (or H2O) is added at t = -25 s (blue arrow) and a negative-inside ΔΨ is established by the addition of valinomycin at t = 0 s (red arrow). No acidification is observed in the absence of DtpT. A slight decrease in fluorescence is observed upon addition of valinomycin in the absence of substrate, likely due to proton leakage. Strong acidification of the lumen indicative of DtpT-mediated transport requires both peptide substrate and ΔΨ. (TIF) [file ppat.1013535.s003.tif]

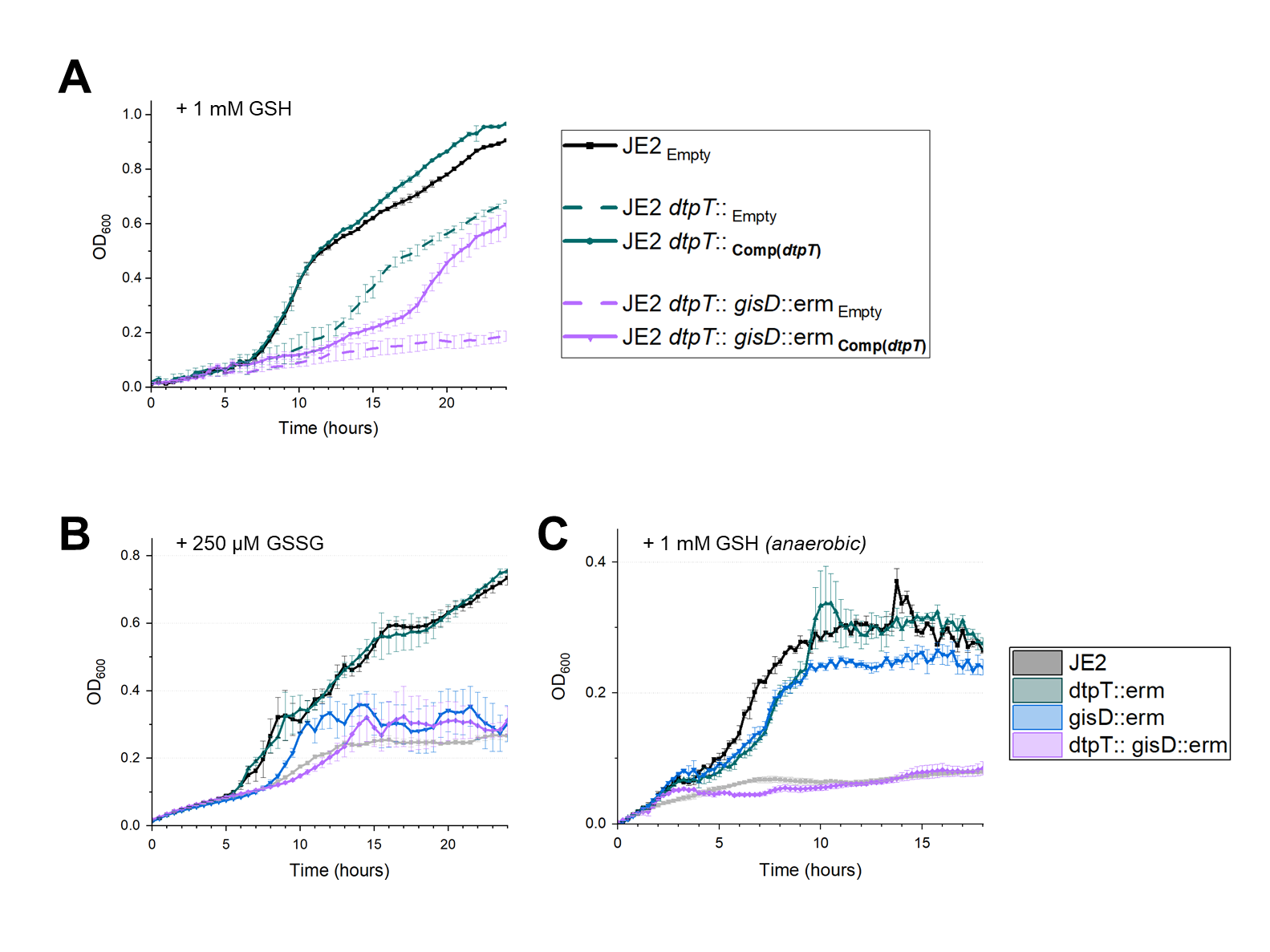

Supplement: S4 Fig — (A) Growth of S. aureus strain JE2 carrying pSK56pT (Empty) and mutant derivatives carrying either pSK56pT or pSK56pTdtpT (Comp) was assessed in CDMG – s supplemented with 1 mM GSH and chloramphenicol. (B - C) Growth of S. aureus strain JE2 and mutant derivatives was assessed in CDMG – s supplemented with 250 µM GSSG (B) in aerobic conditions, as well as in the presence of 1 mM GSH under anaerobic conditions (C). For A and B, strains were grown over 24 hours and OD600 was measured every 30 minutes. For C, strains were grown over 18 hours and OD600 was measured every 15 minutes. Curves indicate the mean values for three biological replicates ± standard deviation. For B and C, a pale grey line indicates equivalent data for strain JE2 in CDMG – s and is included for comparison. (TIF) [file ppat.1013535.s004.tif]

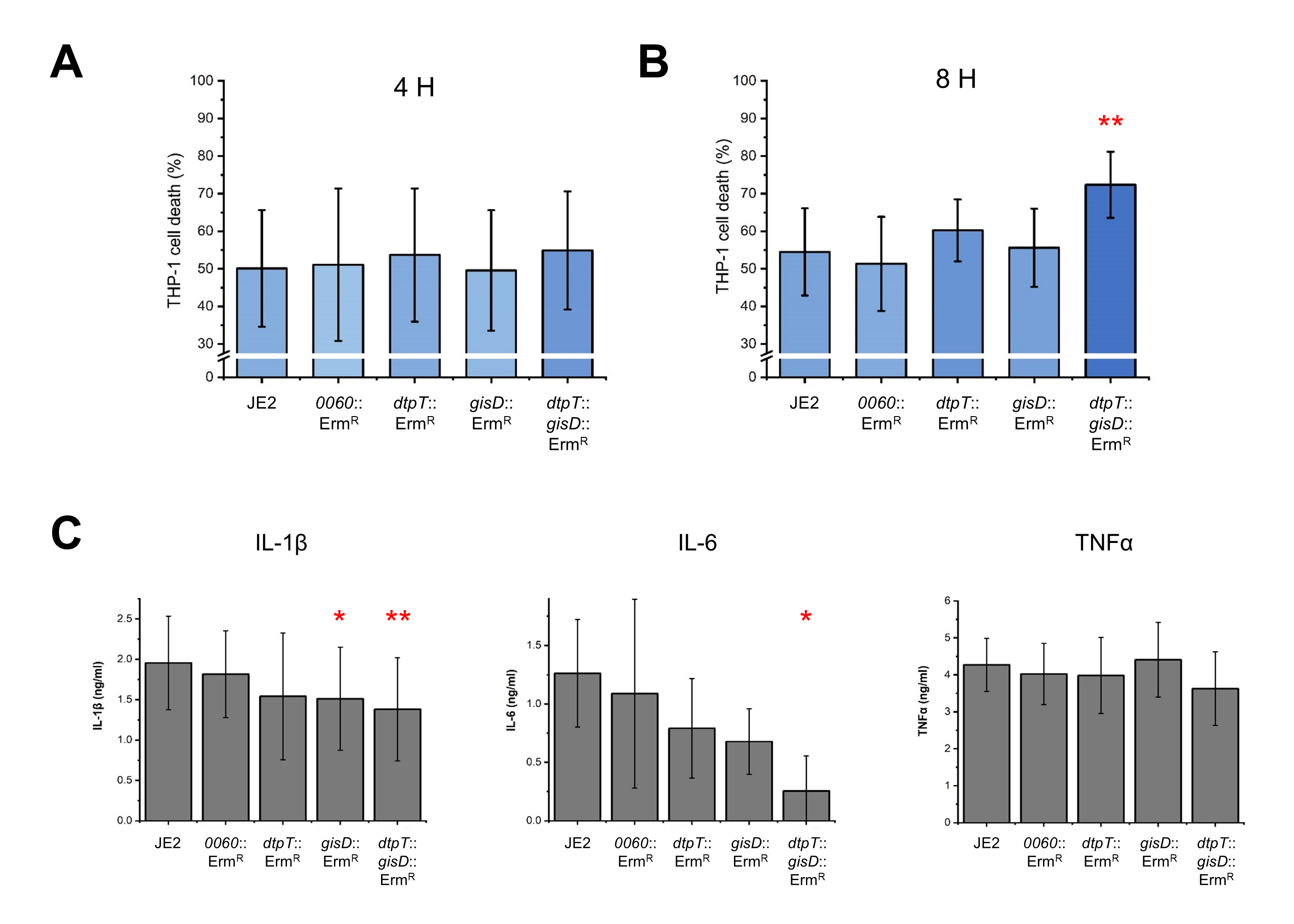

Supplement: S5 Fig — (A - B) Cell death following bacterial infection was quantified at 4 hours (A) and 8 hours (B) post-infection for THP1 cells infected with JE2 or mutant derivatives (n = 6, each measured in technical triplicate). Cell death was quantified by measuring the activity of extracellular LDH in cell culture supernatant. Values are given as a percentage of the LDH activity for cells lysed by addition of 0.04% triton X-100 and corrected for background signal. (C) Production of inflammatory cytokines by infected THP-1 cells at 6 hours post-infection (n = 4, each measured in technical triplicate). *p < 0.05, **p < 0.01; as determined by paired-sample t-test (vs JE2). (TIF) [file ppat.1013535.s005.tif]

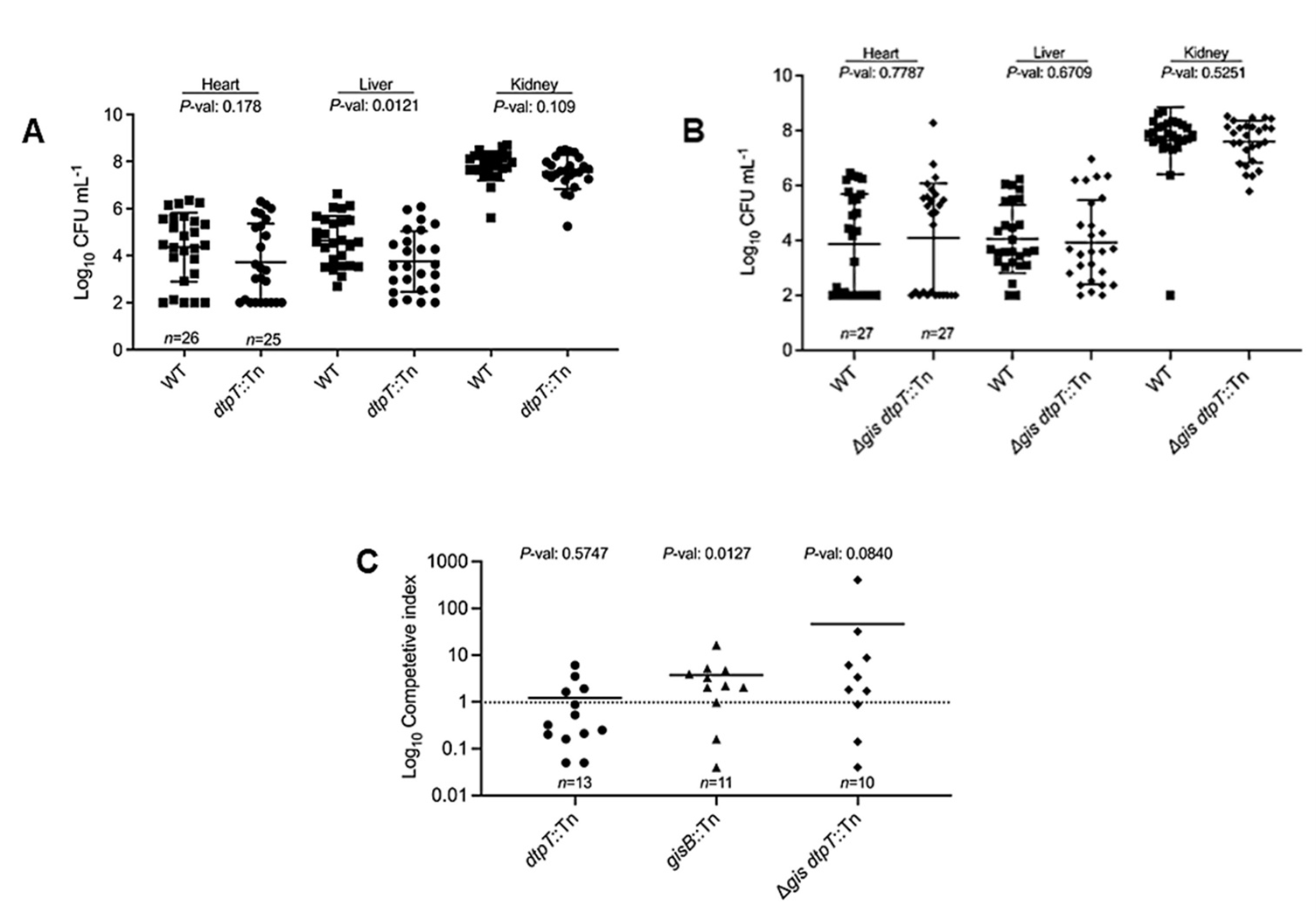

Supplement: S6 Fig — (A - B) Bacterial burdens within indicated organs after systemic inoculation of BALB/cJ mice were enumerated after 96 h of infection with either WT (squares), dtpT::Tn (circles) (A) or ∆gis dtpT::Tn (diamonds) (B). Bacterial burdens are presented as log10 transformed CFUs mL-1 for liver, combined kidneys, and heart. The mean and standard deviation are presented as horizontal lines. Normality was determined using a Shapiro-Wilk test. p values were determined by Mann-Whitney test. (C) Competitive indices (CI) for dtpT::Tn, gisB::Tn, and ∆gis dtpT::Tn were determined from the livers of Balb/cJ mice systemically inoculated with a 1:1 mixture of WT and the indicated mutant strain. The liver output ratio of WT to indicated mutant CFU mL-1 in the liver over the input WT to mutant CFU mL-1 were used to calculate the CI. The mean of WT:indicated mutant CI are presented as a horizontal bar and p values denoted for each competition were determined by Wilcoxon signed-ranked test. (TIF) [file ppat.1013535.s006.tif]

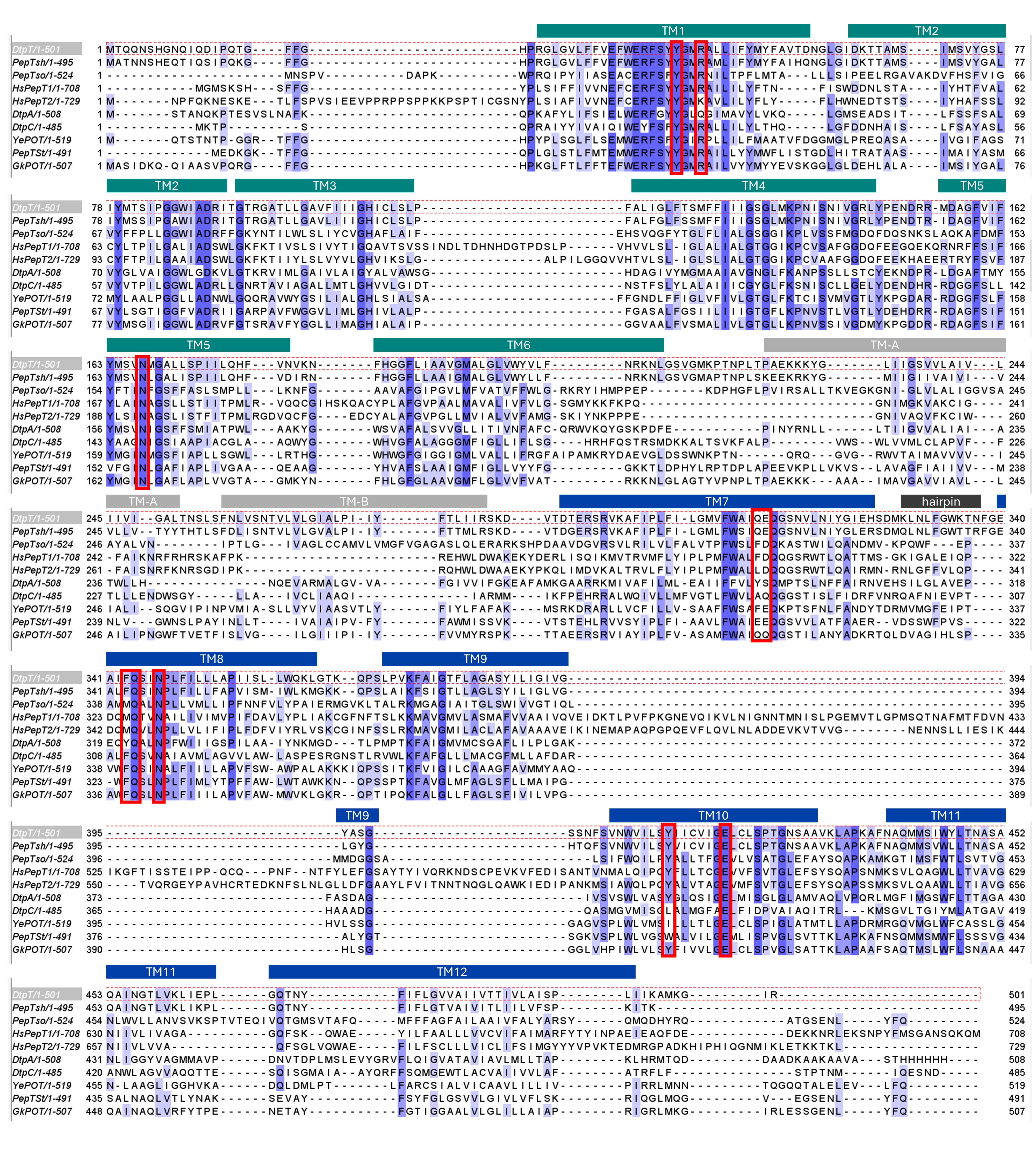

Supplement: S7 Fig — Multiple protein sequence alignment was carried out by Clustal Omega [Madeira et al., 2024] and visualised in Jalview [Waterhouse et al., 2009]. The sequence of DtpT (from S. aureus JE2) is highlighted. Sequences are given in FASTA format and coloured by identity (blue). Residues predicted to contribute toward GSH binding in DtpT are highlighted by red boxes. Regions corresponding to predicted structural elements in DtpT are also labelled above each line. (TIF) [file ppat.1013535.s007.tif]

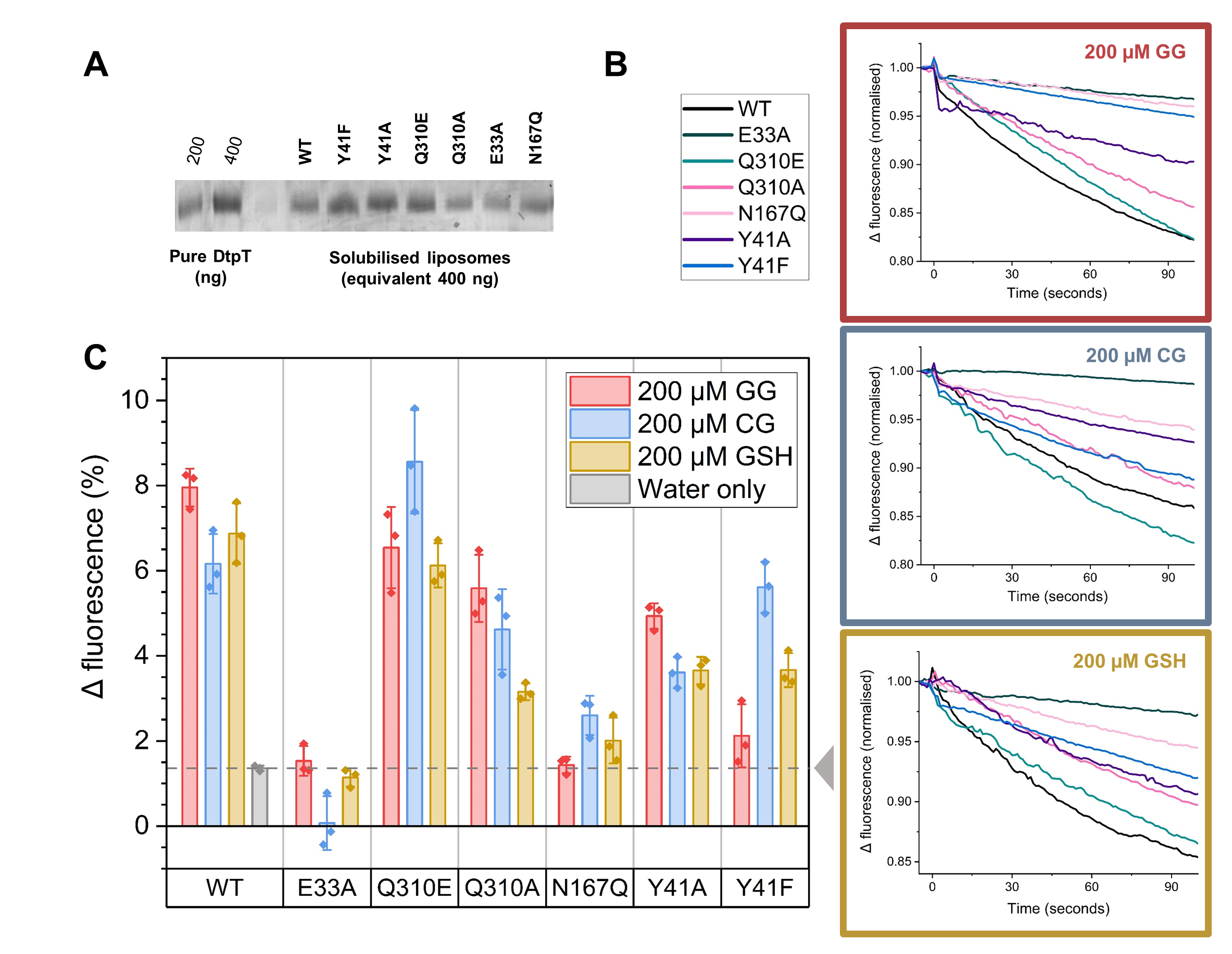

Supplement: S8 Fig — (A) SDS PAGE comparison of pure DtpT against solubilised liposomes containing wild-type or mutant variants of DtpT, as labelled. Band intensities were used to normalise the final liposomal protein concentration to 0.5 mg/ml. (B) Complete transport assay data for each of three peptide substrates, normalised immediately before the addition of valinomycin for ease of comparison. Curves show the mean of three replicates. (C) Summarised transport data for DtpT and mutant variants against gly-gly, cys-gly and GSH. Transport activity is compared by quantifying the change in pyranine fluorescence over the first 30 seconds of the assay. A grey dashed line indicates the Δ fluorescence (%) recorded for DtpT liposomes in the absence of substrate (water only). Bars indicate the mean ± standard deviation. (TIF) [file ppat.1013535.s008.tif]
