# Supplementary material for: Defined roles for the Staphylococcus aureus POT transporter DtpT in di/tripeptide uptake and glutathione utilisation inside human macrophages
Source: PLoS Pathog. 2025 Sep 26;21(9):e1013535. doi: 10.1371/journal.ppat.1013535 (PMC12510641; doi:10.1371/journal.ppat.1013535)
Supplement: S1 Table — (PDF) [file ppat.1013535.s009.pdf]

Supplemental table 1. Summary of DtpT substrates identified in this work

Putative DtpT substrates (based on PM assays)					
**Cluster**	**Peptide**	**Cluster**	**Peptide**	**Cluster**	**Peptide**
2*	ala-arg	3	arg-trp	3	thr-asp
2*	ala-asn	3	asn-glu	3	trp-glu
2*	arg-ala	3	asp-ala	3	tyr-glu
2*	arg-arg	3	asp-asp	3	val-glu
2*	arg-gln	3	asp-gln	3	val-gly
2*	arg-ile	3	asp-leu	4	ala-gly
2*	arg-leu	3	asp-phe	4	ala-leu
2*	arg-lys	3	asp-val	4	ala-lys
2*	arg-met	3	glu-ala	4	asn-val
2*	arg-ser	3	glu-asp	4	asp-gly
2*	arg-tyr	3	glu-gly	4	asp-lys
2*	arg-val	3	glu-ser	4	asp-trp
2*	gly-cys	3	glu-trp	4	b-ala-ala
2*	gly-glu-leu	3	glu-tyr	4	d-ala-gly-gly

2*	gly-gly-gly	3	glu-val	4	d-ala-leu
2*	gly-gly-ile	3	gly-asp	4	glu-glu
2*	gly-gly-phe	3	gly-gly	4	gly-ala
2*	gly-his	3	gly-gly-ala	4	gly-phe-phe
2*	gly-ile	3	gly-met	4	gly-tyr
2*	gly-leu	3	gly-phe	4	his-gly
2*	ile-arg	3	gly-pro	4	his-pro
2*	ile-asn	3	gly-ser	4	ile-ala
2*	ile-leu	3	gly-val	4	ile-gly
2*	ile-ser	3	his-glu	4	ile-ile
2*	leu-arg	3	leu-asp	4	ile-pro
2*	leu-asn	3	leu-glu	4	ile-val
2*	leu-gly-gly	3	leu-pro	4	leu-ala
2*	leu-ile	3	leu-val	4	leu-b-ala
2*	leu-leu	3	lys-asp	4	leu-gly
2*	leu-leu-leu	3	lys-glu	4	leu-met
2*	leu-phe	3	lys-gly	4	lys-pro
2*	lys-arg	3	lys-leu	4	lys-ser
2*	phe-gly-gly	3	met-arg	4	lys-val

2*	pro-arg	3	met-glu	4	met-asp
2*	pro-ile	3	met-gly	4	met-his
2*	pro-leu	3	phe-asp	4	met-leu
2*	pro-lys	3	phe-glu	4	met-thr
2*	pro-trp	3	phe-gly	4	phe-ala
2*	ser-asn	3	phe-pro	4	pro-glu
2*	ser-phe	3	pro-asn	4	ser-his
2*	val-asn	3	pro-gly	4	thr-glu
3	ala-asp	3	pro-phe	4	val-asp
3	ala-glu	3	ser-ala	4	val-ile
3	ala-ser	3	ser-asp	4	val-leu
3	arg-asp	3	ser-glu	4	val-pro
3	arg-glu	3	ser-gly	4	val-val
3*	arg-phe	3	ser-pro		

Confirmed DtpT substrates

Cluster	Peptide	Cluster	Peptide	Cluster	Peptide
7	ala-phe	3	met-gly	7	ala-ala-ala

3*	arg-phe	4	ala-gly	3	gly-pro
2*	leu-gly-gly	4	ala-leu	3	gly-gly
3	gly-phe	7	cys-gly	7	ala-ala
2*	gly-gly-gly	2*	arg-ala	N/A	GSH
3	gly-asp	3	arg-asp		
1	ala-gln	N/A	gly-glu		

* Utilisation pattern suggests that these peptides are also utilised via Opp3.
