# Supplementary material for: Defined roles for the Staphylococcus aureus POT transporter DtpT in di/tripeptide uptake and glutathione utilisation inside human macrophages
Source: PLoS Pathog. 2025 Sep 26;21(9):e1013535. doi: 10.1371/journal.ppat.1013535 (PMC12510641; doi:10.1371/journal.ppat.1013535)
Supplement: S2 Table — (DOCX) [file ppat.1013535.s010.docx]

**Supplemental table 2. Strains utilised in this work**

| **Strain** | **Organism** | **Background** | **Relevant genotype** | **Notes** | **Relevant source** |
| --- | --- | --- | --- | --- | --- |
| **IK5** | *S. aureus* | JE2 |  |  | Fey et al., 2013 |
| **IK2** |  |  | *dtpT*::Erm^R^ | Generated via Φ85 phage transduction from NTML strain NE971 into *IK5* | Fey et al., 2013 |
| **IK163** |  |  | *opp3A*::Erm^R^ | Generated via Φ85 phage transduction from NTML strain NE440 into *IK5* | Fey et al., 2013 |
| **IK38** |  |  | *dtpT::* | *ermR* casette cleared from *bursa aurealis* via allelic exchange | Bose et al., 2013 |
| **IK165** |  |  | *dtpT*:: *opp3A*::Erm^R^ | Generated via Φ85 phage transduction from NTML strain NE440 into *IK38* | Fey et al., 2013 |
| **GHT2288** | *E. coli* | DH5α | pSK5630 | *E. coli* / *S.aureus* shuttle vector | Grkovic et al., 2003 |
| **IK161** | *E. coli* | DH5α | pSK56_pT_ | 1080 bp of sequence immediately upstream of *dtpT* in the *S. aureus* JE2 genome (incorporating the DtpT promoter sequence) was cloned into pSK5630 | This study |
| **IK170** |  |  | pSK56_pT_*dtpT* | The entire *dtpT* open reading frame and 1080 bp of upstream sequence from *S. aureus* JE2 was cloned into pSK5630 | This study |
| **IK12** | *S. aureus* | RN4220 |  | Cloning intermediate strain | Kreiswirth et al., 1983 |
| **IK176** | *S. aureus* | JE2 | pSK56_pT_ |  | This study |
| **IK177** | *S. aureus* | JE2 | *dtpT::* + pSK56_pT_ |  | This study |
| **IK204** |  |  | *dtpT::* + pSK56_pT_*dtpT* |  | This study |
| **IK209** | *S. aureus* | JE2 | *dtpT*:: *opp3A*:: Erm^R^ + pSK56_pT_ |  | This study |
| **IK212** |  |  | *dtpT*:: *opp3A*::Erm^R^ + pSK56_pT_*dtpT* |  | This study |
| **IK208** | *S. aureus* | JE2 | *gisD*::Erm^R^ | Generated via Φ85 phage transduction from NTML strain NE215 into *IK5* | Fey et al., 2013 |
| **IK207** |  |  | dtpT:: *gisD*::Erm^R^ | Generated via Φ85 phage transduction from NTML strain NE215 into *IK38* | Fey et al., 2013 |
| **IK213** | *S. aureus* | JE2 | *dtpT*:: *gisD*:: Erm^R^ + pSK56_pT_ |  | This study |
| **IK214** |  |  | *dtpT*:: *gisD*::Erm^R^ + pSK56_pT_*dtpT* |  | This study |
| **IK206** | *S. aureus* | JE2 | SAUSA300_0060  ::Erm^R^ | Generated via Φ85 phage transduction from NTML strain NE806 into *IK5* | Fey et al., 2013 |
| **IK32** | *E. coli* | BL21(DE3) | pWaldo | *E. coli* membrane protein expression vector containing C-terminal octa-His GFP-tag | Drew et al., 2006 |
| **IK81** | *E. coli* | C43 |  |  | Miroux and Walker, 1996 |
| **IK98** | *E. coli* | C43 | pWaldo-DtpT |  | This study |
| **IK187** |  | C43 | pWaldo-DtpT_Y41F_ |  | This study |
| **IK188** |  |  | pWaldo-DtpT_Y41A_ |  | This study |
| **IK191** |  |  | pWaldo-DtpT_Q310E_ |  | This study |
| **IK197** |  |  | pWaldo-DtpT_Q310A_ |  | This study |
| **IK189** |  |  | pWaldo-DtpT_E33A_ |  | This study |
| **IK190** |  |  | pWaldo-DtpT_N167Q_ |  | This study |

**S2 References:**

1. Bose, J. L., Fey, P. D., & Bayles, K. W. (2013). Genetic tools to enhance the study of gene function and regulation in Staphylococcus aureus. Applied and Environmental Microbiology, 79(7), 2218–2224.
2. Drew, D., Lerch, M., Kunji, E., Slotboom, D.-J., & de Gier, J.-W. (2006). Optimization of membrane protein overexpression and purification using GFP fusions. Nature Methods, 3(4), 303–313.
3. Fey, P. D., Endres, J. L., Yajjala, V. K., Widhelm, T. J., Boissy, R. J., Bose, J. L., & Bayles, K. W. (2013). A genetic resource for rapid and comprehensive phenotype screening of nonessential Staphylococcus aureus genes. MBio, 4(1), e00537-12.
4. Grkovic, S., Brown, M. H., Hardie, K. M., Firth, N., & Skurray, R. A. (2003). Stable low-copy-number Staphylococcus aureus shuttle vectors. Microbiology, 149(Pt 3), 785–794.
5. Kreiswirth, B., Löfdahl, S., Betley, M., O’Reilly, M., Schlievert, P., Bergdoll, M., & Novick, R. (1983). The toxic shock syndrome exotoxin structural gene is not detectably transmitted by a prophage. Nature, 305, 709–712.
6. Miroux, B., & Walker, J. E. (1996). Over-production of proteins in Escherichia coli: mutant hosts that allow synthesis of some membrane proteins and globular proteins at high levels. Journal of Molecular Biology, 260(3), 289–298.
