# Supplementary material for: Defined roles for the Staphylococcus aureus POT transporter DtpT in di/tripeptide uptake and glutathione utilisation inside human macrophages
Source: PLoS Pathog. 2025 Sep 26;21(9):e1013535. doi: 10.1371/journal.ppat.1013535 (PMC12510641; doi:10.1371/journal.ppat.1013535)
Supplement: S3 Table — (DOCX) [file ppat.1013535.s011.docx]

**Supplemental table 3. Primers utilised in this work**

| **Primer number** | **Target** | **Sequence** | **Function** |
| --- | --- | --- | --- |
| 1 | *dtpT* flanking regions | GGGCAAGTTCACACCACG | diagnostic PCR |
| 2 |  | GTTAACCGTGCCATCACCG |  |
| 3 | *opp3A* flanking regions | CCTTAGCTCCTGAAAATCTCGTCG | diagnostic PCR |
| 4 |  | GCGGATTTGAACGTTGCGAAG |  |
| 5 | *gipD* flanking regions | GGTGTTTGTATTATGTTCGTCG | diagnostic PCR |
| 6 |  | TGGTTCTAACACATTCAATGCC |  |
| 7 | *Bursa aurealis* Tn | GCTTTTTCTAAATGTTTTTTAAGTAAATCAAGTAC | diagnostic PCR |
| 8 | pWaldo | GAAGCAGCCCAGTAGTAGG | diagnostic PCR/sequencing |
| 9 |  | GTGTTGGCCATGGAACAGG |  |
| 10 | pSK5630 | CCTTAGCTCCTGAAAATCTCGTCG | diagnostic PCR/sequencing |
| 11 |  | GCGGATTTGAACGTTGCGAAG |  |
| 14 | pWaldo cloning site | GTCGAGTCTCCTTCTTAAAGTTAAAC | vector linearisation |
| 15 |  | GAAAACCTGTACTTCCAGGGTCA |  |
| 16 | pSK5630 cloning site | GATCCGTCGACCTGCAGC | vector linearisation |
| 17 |  | CCCTGGCAGTTTATGGCGG |  |
| 20 | *dtpT* | (TAACTTTAAGAAGGAGACTCG)ACATGACACAACAAAACTCCCA | Cloning into pWaldo |
| 21 |  | (CCTGGAAGTACAGGTTTTC)ACGTATACCTTTCATCGCTTTGA |  |
| 22 | *dtpT* | (CCGCCATAAACTGCCAGGG)ATTAACGTATACCTTTCATCGC | cloning into pSK5630 |
| 23 | *dtpT* promoter region | (GCTGCAGGTCGACGGATC)GGGTCAAATTGGTGTTACTT |  |
| 24 |  | (CCGCCATAAACTGCCAGGG)CATGTATACATCCCATCCTTTC |  |
| 27 | *dtpT* | CGTACCAATTGCAAATTTTACTGG | diagnostic PCR |
| 28 | *dtpT* | CTGGGCTATTgcaGAACAAGGGTC | Q310A mutation |
| 29 |  | CTGGGCTATTgaaGAACAAGGGTC | Q310E mutation |
| 30 |  | AACACCATTCCAAGAATAAATAATG | Q310 reverse |
| 31 |  | GTTTAGTTATtttGGCATGCGTG | Y41F mutation |
| 32 |  | GTTTAGTTATgctGGCATGCGTGC | Y41A mutation |
| 33 |  | CTTTCCCAGAACTCTACAAAG | Y41 reverse |
| 34 |  | CTTCTTTGTAgcgTTCTGGGAAAG | E33A mutation |
| 35 |  | AGTACGCCTAGTCCTC | E33 reverse |
| 36 |  | TATGTCAGTTcaaATGGGTGCATTATTATC | N167Q mutation |
| 37 |  | TAGAAAATAACAAAACCTGC | N167 reverse |
