# Supplementary material for: Defined roles for the Staphylococcus aureus POT transporter DtpT in di/tripeptide uptake and glutathione utilisation inside human macrophages
Source: PLoS Pathog. 2025 Sep 26;21(9):e1013535. doi: 10.1371/journal.ppat.1013535 (PMC12510641; doi:10.1371/journal.ppat.1013535)
Supplement: S4 Table — (DOCX) [file ppat.1013535.s012.docx]

**Supplemental table 4. Composition of CDMG (adapted from Hussain et al., 1991)**

| **Ingredients** | **mg/L** |  | **Ingredients** | **mg/L** |
| --- | --- | --- | --- | --- |
| **Group 1** | **Adjust pH to 7.2, then autoclave in 700 ml** |  | **Group 2** | **Autoclave in 280 ml** |
| Na_2_HPO_4_ | 8000 |  | d-Glucose | 2 000 |
| KH_2_PO_4_ | 3000 |  | MgSO_4_·7H_2_O | 500 |
| l-Aspartic acid | 150 |  |  |  |
| l-Alanine | 100 |  | **Group 3** |  |
| l-Arginine | 150 |  | **Filter sterile to 100x stock** | **+ 10 ml** |
| l-Cysteine | 75 |  | Biotin | 0.1 |
| Glycine | 100 |  | Nicotinic acid | 2 |
| l-Glutamic acid | 150 |  | D-Pantothenic acid, Ca salt | 2 |
| l-Histidine | 100 |  | Pyridoxal | 4 |
| l-Isoleucine | 150 |  | Thiamine hydrochloride | 2 |
| l-Lysine | 100 |  | Pyridoxamine dihydrochloride | 4 |
| l-Leucine | 150 |  |  |  |
| l-Methionine | 100 |  | **Group 4** |  |
| l-Phenylalanine | 100 |  | ***Filter sterile to 100x stock*** | **+ 10 ml** |
| l-Proline | 150 |  | CaCI_2_·6H_2_O | 10 |
| l-Serine | 100 |  | MnSO_4_ | 5 |
| l-Threonine | 150 |  | (NH_4_)_2_SO_4_·FeSO_4_·6H_2_O | 6 |
| l-Tryptophan | 100 |  |  |  |
| l-Tyrosine | 100 |  |  |  |
| l-Valine | 150 |  |  |  |
