# Supplementary material for: Defined roles for the Staphylococcus aureus POT transporter DtpT in di/tripeptide uptake and glutathione utilisation inside human macrophages
Source: PLoS Pathog. 2025 Sep 26;21(9):e1013535. doi: 10.1371/journal.ppat.1013535 (PMC12510641; doi:10.1371/journal.ppat.1013535)
Supplement: S1 File — Supplemental methods supporting S6 Fig. (DOCX) [file ppat.1013535.s013.docx]

#### **SM1 – Supplemental methods supporting figure S6**

**Strains used in animal experiments**

The ∆*gis* and *gisB*::Tn were previously described in Lensmire et al., 2023. The *dtpT*::Tn and ∆*gis dtpT*::Tn strains were generated via phage transduction into the WT JE2 strain background or the previously published ∆*gis* strain background. All strains used in animal experiments are described in the following table:

| **Organism** | **Background** | **Relevant genotype** | **Notes** | **Relevant source** |
| --- | --- | --- | --- | --- |
| *S. aureus* | JE2 |  |  | Fey et al., 2013 |
|  |  | *dtpT*::Tn | Generated via phage transduction from NTML strain NE971 into JE2. Functionally identical to IK2 but generated independently by NDH. | Fey et al., 2013 |
|  |  | *gisB::*Tn | Generated via phage transduction from NTML strain NE215 into JE2. | Fey et al., 2013 |
|  |  | *∆gis* |  | Lensmire et al., 2023 |
|  |  | *∆gis dtpT::Tn* | Generated via phage transduction from NTML strain NE440 into *∆gis* | Lensmire et al., 2023 |

**Single strain murine systemic infections**

WT, *dtpT*::Tn, or ∆*gis dtpT*::Tn mutant strains were cultured in TSB overnight at 37°C, diluted 1:100 into TSB, and cultured for 3 h at 37°C at 225 rpm shaking. Cultures were pelleted, washed with PBS, and normalized to OD_600_ equal to 0.4. Thirty female 8-week-old BALB/cJ mice were retro-orbitally infected with 10^7^ CFUs and the infection proceeded for 96 h after which heart, liver, and kidneys were collected and homogenized in 1 mL PBS. Organ homogenates were serially diluted and plated onto TSA. Bacterial burdens were quantified as CFUs mL^-1^. Infections were performed at Michigan State University under the principles and guidelines described in the Guide for the Care and Use of Laboratory Animals. Animal work was followed as approved by Michigan State University Institutional Animal Care and Use Committee (IACUC) approved protocol number PROTO202200474.

**Murine systemic competition infections**

WT, *dtpT*::Tn, *gisB*:Tn and ∆*gis dtpT*::Tn mutant strains were grown in TSB overnight at 37°C, subcultured 1:100, and grown in TSB for 3 h at 37°C and 225 rpm. Strains were washed in PBS and normalized to an OD_600_ equal to 0.4. The competitions were prepared by mixing equal volumes of WT with the *dtpT*::Tn, *gisB*:Tn or ∆*gis dtpT*::Tn mutant strains. The input ratio was quantified by serially diluted the mixture and plating onto TSA and TSA supplemented with 10 μg mL^−1^ erythromycin (erm^10^) to discern between WT and the mutant strain. The transposon-harboring mutant strains utilized in these experiments harbor an erm resistance cassette within the transposon. WT CFU were calculated by subtracting CFU generated on TSA-erm^10^ from CFU produced on TSA. Fifteen female 8-week-old BALB/cJ mice were retro-orbitally infected with 100 μL containing the mixture of 10^7^ CFU of WT and the indicated mutant strain. After 96 h, the heart, liver, and kidneys were collected and homogenized in 1 mL PBS. Homogenates were serially diluted and plated onto TSA or TSA-erm^10^. Competitive indices were calculated as dividing the WT:mutant strain output CFU ratio by the WT:mutant input CFU ratio.

**Statistical Analysis**

All data were analyzed using GraphPad Prism v9.1.1.

#### **SM1 References:**

1. Fey, P. D., Endres, J. L., Yajjala, V. K., Widhelm, T. J., Boissy, R. J., Bose, J. L., & Bayles, K. W. (2013). A genetic resource for rapid and comprehensive phenotype screening of nonessential Staphylococcus aureus genes. MBio, 4(1), e00537-12.
2. Lensmire, J. M., Wischer, M. R., Kraemer-Zimpel, C., Kies, P. J., Sosinski, L., Ensink, E., Dodson, J. P., Shook, J. C., Delekta, P. C., Cooper, C. C., Havlichek, D. H., Jr, Mulks, M. H., Lunt, S. Y., Ravi, J., & Hammer, N. D. (2023). The glutathione import system satisfies the Staphylococcus aureus nutrient sulfur requirement and promotes interspecies competition. PLoS Genetics, 19(7), e1010834.
