# Supplementary material for: Defined roles for the Staphylococcus aureus POT transporter DtpT in di/tripeptide uptake and glutathione utilisation inside human macrophages
Source: PLoS Pathog. 2025 Sep 26;21(9):e1013535. doi: 10.1371/journal.ppat.1013535 (PMC12510641; doi:10.1371/journal.ppat.1013535)
Supplement: Attachment — Submitted filename: Response to reviewers for Imran PLOS Pathogens.docx [file ppat.1013535.s019.docx]

Department of Biology

YO10 5YW, York, UK

Prof. Gavin H. Thomas

E-mail: gavin.thomas@york.ac.uk

Prof. Andreas Peschel,

Academic Editor,

PLOS Pathogens

21^st^ August 2025

Dear Prof. Peschel,

Many thanks for handling our submission and for the reviewers’ comments that we have been acting on in the last month or so. We have added additional experimental data (Fig. 5C) which we think addressed the major scientific question raised by reviewer 1, and explained, we hope to your satisfaction, how the Biolog assays work more clearly in terms of them not being ‘growth’ assays and how robust controls and optimisation of the setup enabled us to be confident of the trends in the data we obtained (of which important ones were tested using independent methods subsequently in the paper). We hope these additional experiments, corrections and clarifications are sufficient to make the paper acceptable in your journal.

Sincerely,

Gavin H. Thomas (on behalf of the authors)

Overall, we thank the two reviewers for their overall positive assessment of our work and in the following pages outline our revisions we have made to the manuscript to address their specific comments.

**Part II – Major Issues: Key Experiments Required for Acceptance**

Reviewer #1:

**Comment 1.** One of the major aspects of the manuscript is the documentation that DtpT transports glutathione but is not the major glutathione transporter (GisD). In these cases where the transporters are known, transport assays should be performed to understand the transport kinetics to more fully understand the hypothesis that GisD is a high affinity transporters whereas DtpT is a low affinity transporter (similar to Figure 7E). This would add significant rigor to the manuscript.

This is an important point that we have been happy to undertake additional experimentation to demonstrate in the revised manuscript. While we have an *in vitro* system for DtpT, which has taken some years to work-up with expert collaborators at the University of Oxford, having the same for a complete bacterial uptake ABC transporter (multi-subunit) is no trivial undertaking and could take a huge amount of additional work. However, to address the important point made by the reviewer, we have taken a widely used *in vivo* approach to address the same biological question and have added some strong additional data to support the idea of the differential affinities of the two transport systems (see Fig. 5C). In this experiment we have determine the overall K_S_ of whole cells for glutathione, as uptake is usually the rate limiting step for growth, and used our four strains with all, one or neither of the transporters present, in growth conditions where we have varied the concentration of glutathione (GSH) as the sole nitrogen source.

The patterns of growth demonstrate very clearly that growth a low micromolar concentrations of GSH requires the Gis ABC transporter and having DtpT alone cannot support growth, while at concentrations from 500 uM GSH and above, the *gisD* mutant can now start to grow, with growth almost identical to the wild-type strain at 2.5 mM GSH, demonstrating the role for DtpT at higher concentrations. Based on these data, we conclude that Gis operates with a higher affinity for GSH than DtpT and is, therefore, likely to be more relevant for GSH acquisition in GSH-depleted environments.

**Reviwer 1, Comment 2**: Unclear if the last part of the study regarding Listeria should be added to the manuscript. Maybe this should be discussed in the discussion section?

We agree with the reviewer that the analysis referenced here is probably more suited to the discussion than results. We have implemented this change in the manuscript.

**Reviewer 1, Comment 3**: Although the assay seems to have worked, the Biolog assays are difficult to interpret at best as one does not know what types of compounds are in the wells (proprietary information--they were designed for prototrophs). For instance, as noted by the authors, S. aureus requires a significant number of amino acids for appropriate growth so nitrogen based assays are difficult to design. I am unsure what the authors can say about this as they confirmed many of the results, but, again, difficult to interpret as you can't design how the assay is run.

Please see a full discussion of the pros- and cons of the Biolog approach in the answer to Reviewer 2 Comment 1.

**Reviewer 2, Comment** 1: The authors used a commercially available phenotype microarrays (“Biolog Plates”). S. aureus however exhibits distinct metabolic peculiarities such as auxotrophies, genetic and regulatory ones, that set it apart from the model organism E. coli. These auxotrophies make conclusions of nutrient or nitrogen metabolization difficult since S. aureus metabolism has many unresolved interdependencies as certain amino acids and cofactors have to be included into minimal growth media (see Audretsch et al 2021, https://doi.org/10.1038/s41598-021-88646-1, and Machado et al 2019, https://doi.org/10.1128/AEM.01773-19). These essential growth factors often contain nitrogen themselves and could potentially be utilized as direct nitrogen source? Only cysteine was apparently added. What about other essential aminoacids? Hence several essential growth factors (amino acids or cofactors such as thiamine) can bottleneck quantifiable growth and not the supplied peptides themselves. Data in the manuscript that addresses the applicability or usability of commercial phenotype microarrays for S. aureus to extract high quality scientific results does not appear in the manuscript.

Here, both reviewers correctly identify that *S. aureus* should not be able to grow strongly under phenotype microarray (PM) conditions due to the lack of essential nutrients, such as amino acids for which the organism is auxotrophic as well as vitamins including thiamine and biotin. Indeed, these nutrient dependencies are well documented and accounted for in growth assays presented later throughout this study (for instance, in Figure 3E or Figure 5C/D). However, the inclusion of these additional nutrients (specifically, amino acids) in the PM assay would provide the cells with additional nitrogen sources, which would likely mask the peptide-dependent utilisation phenotypes we hoped to demonstrate here.

The important point here is that the PM assay does not utilise bacterial cell density (growth) as the indicator of nutrient utilisation, and this is a notable distinction between this assay and the aforementioned growth assays. As described in the text (starting line 149), this assay instead relies on a highly sensitive tetrazolium dye to detect nitrogen source utilisation. To expand on this; cells are supplied with exogenous sources of both carbon (glucose/pyruvate) and sulphur (cystine; also serving as an oxidising buffer to prevent spontaneous dye reduction) while nitrogen availability is limited primarily to the supplied peptide in each well. Both transport and catabolism of the peptide are required to yield nitrogen, which is used to drive cellular metabolic processes which result, ultimately, in the synthesis of NADH. Electrons from NADH are transferred to the tetrazolium dye, thereby reducing the dye and producing a purple colour which is quantified colorimetrically over time resulting in a signal curve (described further by Bochner et al., 2001). In other words, conversion of the dye represents the cellular metabolic output under conditions where respiration is strictly dependent on efficient nitrogen source utilisation.

Empirically, we can confirm that the PM assay results are a valid indicator of nitrogen source utilisation as each plate includes two important controls: L-glutamine in well A2 (positive) and no nitrogen source in well A1 (negative). Before conducting the PM assays, we carried out a series of optimisation screens in which the initial bacterial inoculum and carbon source concentrations were varied to ensure a clear and consistent distinction between the signal curves observed for these control conditions. These efforts resulted in the conditions utilised in our study, as described in the “methods” section. Under these conditions, we observed a robust colorimetric signal in the positive control wells across all experiments, while the negative controls consistently gave a far weaker signal (residual signal is presumed to be background, resulting from trace metabolic activity in the absence of a nitrogen source). The data for these controls are available in supplemental datasets 1-4 and can be seen in the raw data. We believe that these controls effectively validate our PM data in two important ways. Firstly, they demonstrate that a strong colorimetric signal output requires the presence of a suitable nitrogen source. Secondly, these controls demonstrate that a positive signal in this assay does not require supplementation with any additional amino acids or nutrients beyond those already supplied.

Bacterial growth under PM conditions is expected to be strictly limited. Indeed, the PM signal curves presented here track the reduction of the tetrazolium dye (detected at λ = 590 nm) and are corrected for any changes in bacterial cell density (detected at λ = 750 nm) at each time point. The complete OD590 and OD750 value recorded are available in the “raw data to support figures” file. Inspecting these OD750 values, we observe strictly limited growth under all conditions, with marginally increased growth in some wells seemingly dependent on the availability of peptide-derived arginine. These data support the reviewer’s observations, but ultimately this represents a secondary output for the assay and does not offer additional insight into peptide utilisation beyond what is already indicated by the overall signal curves.

Finally, we feel it is important to note that the use of phenotype microarrays as a method of investigating *S. aureus* physiology has already been well established in the literature (see references below) and these earlier studies demonstrate the applicability of the approach for understanding metabolic processes in this organism.

Sources:

Von Eiff et al., 2006 - 10.1128/JB.188.2.687-693.2006

Dunkley et al., 2019 - 10.1371/journal.pone.0219879

Coll et al., 2025 - 10.1038/s41467-024-55186-x

Spreaker et al., 2024 - 10.1111/mmi.15231

**Reviewer 2, Comment 2**: Furthermore omission of certain aminoacids which essential due to repression by Ccpa or CodY often lead to the creation of suppressor mutants. Those suppressor mutant however often have brought effects potentially on other systems such as dtpT or opp3A. Appearance of suppressor mutants usually can be detected and excluded based on their stochastic appearance. These points bring me to the following questions which I feel the authors should address. In general I think the quality of scientific information of the screening approach is strongly overemphasized. I don’t find any biological or technical replicates mentioned by the authors used Biolog plates screen. How many were used for the screen? Were potential suppressor mutants overserved and excluded?

Conventionally (and according to the manufacturer’s supplied protocols), the use of Biolog PMs for phenotypic screening is limited to a single-run experiment. Indeed, the plates required for replicate experiments are prohibitively costly. Hence, the PM assay data presented in this study represents a single biological/technical replicate for each strain and plate combination (i.e. 12 plates total). In spite of this – and while we understand the reviewer’s concerns regarding the possible impact of stochastic effects on our results – we do not believe that such effects meaningfully detract from the key conclusions drawn from the assay, i.e. the detection of overall trends in amino acid use which are later validated by independent methods.

.

Here, our aim was to carry out a preliminary screen for peptide utilisation phenotypes across our wild-type JE2 and transporter-deficient mutant derivative strains, with the primary goal of identifying DtpT-dependant utilisation phenotypes which we could then validate biochemically and through additional growth experiments. From our PM assay, we identified seven distinct phenotypic patterns (figure 1) with clear amino acid-specificity trends apparent across these phenotypic patterns (figure 3). These trends represent the major take-away from the PM assay, highlighting how distinct peptide transporters may allow S. aureus to access specific amino acid nutrients.

The aforementioned amino acid-specific trends are highly robust, as validated by our statistical analysis. All trends presented in figure 3 are associated with a q score of < 1*10-4 according to a Fisher exact test, meaning that the likelihood of these patterns arising due to random/stochastic events is near-zero. To further validate our findings, we also utilise growth assays to confirm DtpT-specific utilisation of three Arg-containing peptides identified as putative DtpT substrates in our PM experiments (figure 3E) and further validate DtpT-specific transport of 12 putative substrates (including the three Arg-containing peptides) via biochemical assays (figure 4). These endeavours help to reinforce the validity of our findings and provide further clarity into the transporter-specific interactions observed.

Regarding suppressor mutations; as already mentioned, PM assays are conducted under conditions wherein growth of cells is strictly limited. Instead, the phenotypic response provides a measure of the total metabolic output of the cells present in each well. Based on this, we predict that the appearance of random suppressor mutants in the cell population will have a minimal effect on the phenotypic responses measured. Indeed, such stochastic effects do not seem to detract from the ability to observe meaningful trends in the data (as outlined above).

**Reviewer 2, comment 3**: From the description in the method section the Biolog plates contained the amino acid cystein which can be essential in S. aureus. However several other amino acids are also essential under certain regulatory regimens. Was this tested in the setup of the screen? Also 3 different carbon sources were used in the growth medium used for the screening: pyruvate, glucose, and citric acid. Previous work showed strong effect of supplemented carbon sources on the ability to synthesize amino acids (Lit et al 2010, doi:10.1128/JB.00237-10). Have the authors considered these effects?

Regarding carbon sources: in order to avoid the need for further optimisation steps, we chose to use a 1:2 mixture of glucose and pyruvate as carbon sources in the PM assay. The inclusion of glucose and pyruvate in the assay is intended to provide cells with rich carbon sources to drive cellular metabolism in the presence of an available nitrogen source. In addition, a high concentration of tricarballylic acid (a structural analogue of citrate) is included to inhibit the enzyme aconitase and thereby disrupt progression through the Krebs’ cycle, limiting total respiratory activity and thereby improving the sensitivity of the assay.

We concede that these carbon sources will likely impact bacterial physiology in regard to nitrogen source utilisation (indeed, as the reviewer points out, previous evidence demonstrates how amino acid metabolic pathways are specifically modulated by carbon source availability through regulators including as CcpA). However, the inclusion of carbon sources is required to enable cellular respiration and, therefore, conversion of the tetrazolium dye as part of the assay. While other carbon sources may have been considered instead, these would likely have had their own distinct phenotypic consequences in regard to nitrogen source utilisation. Ultimately, given that transporter-specific phenotypes were seemingly apparent from our PM dataset, we saw no reason to modify the protocol in this regard. As already mentioned, the validity of these assays is reinforced by the inclusion of robust controls, alongside the robust statistical significance of the trends observed and thorough validation.

**Reviewer 2, comment 4**: The authors used an unsupervised clustering algorithm for the obtained growth curves. Usually this kind of analysis should be performed if the content of the dataset is of high complexity and difficult to interpret with classical methods. I think this is not the case as the datasets seems to consist of clear growth or weak/no growth events. The authors should explain why they used K-means clustering with the K parameter set to k=7. In the end the algorithm just “presses” the data into 7 arbitrary bins. Why not k=3 or k=30? The authors make it seems that there are 7 observable distinct phenotypic outcomes which is an artifact of their data analysis pipeline. The K-means clustering already quite strongly hints that each growth curve can be interpretated directly and more simply by the respective growth values....

I would suggest approaching the screening data more actually based on the observed phenotypes from the growth curves. Each growth curve would here be classified as “no/ growth” or growth/strong growth based on the actual control datapoints (DMSO and glutamine) included in the Biolog plates. (The controls are also not shown/addressed by the authors). Hence each supplied peptide substrate can than be binned into a certain group based on the phenotypic “growth fingerprint”.

We understand the reviewer’s concerns regarding the analytical approaches utilised to process our PM assay data, and apologise that the full justification for the described computational approach (including the use of k=7 clusters) was not included in the original manuscript. This is now expanded upon in the associated methods section.

To clarify; initially, our analysis of the PM data was limited to manually assigned phenotypic groupings based on comparisons between each signal curve and the positive/negative controls, as described here by the reviewer. One major take-away from these methods was the identification of seven common strain-specific patterns in the data, each consistent with a distinct peptide utilisation phenotype. However, we ultimately were dissatisfied with this manual approach for a number of reasons (outlined below), prompting us to utilise an unsupervised clustering approach as an alternative. By applying a k-parameter of K=7, this computational approach was independently able to recreate the seven groupings we had already identified, and coupling this method with UMAP reinforced that each cluster corresponds with a distinct grouping of related data points (indicated by the close spatial organisation of each cluster on the UMAP plot).

There are a few reasons why we were ultimately unsatisfied with our initial bulk analysis. Firstly, a manual binning approach requires that the signal curve data for each condition is reduced to a single numerical value taking into account the total shape of the data for each well. For this purpose, we calculated the empirical area under the curve (eAUC) for each signal curve, and these values are presented in figure 1D. Though eAUC provides a good approximation of the overall strength of the phenotypic response, inspection of the complete signal curve data (supplemental datasets 1-3) reveals heterogeneity in the shape of these signal curves, including differences in the gradient and signal maxima, and these differences may not be effectively captured by a single metric such as eAUC. Ultimately, we decided that the data for each peptide was too complex to be reduced to a single metric.

Additionally, manual binning requires the establishment of clear cut-off points representing positive, negative and intermediate signal values. While our positive/negative controls provide a good guideline for this, we noted a number of results with values close-but-outside of these control values. How do we decide where to draw the boundaries between categories without exerting personal bias in our perception of the data? For instance, a strong positive-like signal curve is observed for JE2 in the presence of Gly-Pro, indicative of utilisation. However, the eAUC value for JE2/Gly-pro was recorded as 15.88; far exceeding the mean negative control value of 6.2, but not quite reaching the mean positive control value of 18.0. An unsupervised computational approach is effective for categorising such corner-cases systematically and independently of our own impressions of the data.

Inevitably – whether utilising manual or computational methods – clustering the data into a limited number of discrete categories necessitates a reduction in the complexity of the data, meaning some of the minutia of individual data points are not fully represented in the final trends presented. For this reason, we also included the signal curves and eAUC values for each strain/peptide combination in the supplemental material (SD1-4), so that interested readers can more closely scrutinise the phenotypic patterns associated with each individual peptide.

**Reviewer 2, comment 5**: Data presentation is often not clear or consistent. Often figure legends are lacking key information. I suggest to improve the way the data is presented by using clear consistent rules for presenting the data. Figure 1 and 3 show barplots with the same set of strains but are colours not used consistently in both figures. Figure 2 uses a lot of colours to highlight the 7 k clusters. However the used palette does not appear very suitable for people with impaired colour perception. The authors should use pallets or contrasts that are suitable for people with colour vision deficiency.

We agree with the reviewer that the use of colour among barplots should be standardised, and this change has been implemented.

Regarding the colour palette used for the seven k clusters across figures 1-4; this colour palette was chosen specifically to facilitate readability by GHT, who is red/green colour-blind. That being said, we had not considered other colour vision deficiencies when constructing these figures, specifically figure 2. This figure has been updated with improved labelling and each cell of the grid has been overlayed with the corresponding k cluster number.

**Reviewer 2, comment 6**:I further recommend summarizing the information of the different assays performed in regards to the substrate specificity of DtpT in a table. This could be a good resource for further studies on the system.

We agree that this will provide a useful resource for future studies. The results of the PM assay and follow-up growth/biochemical experiments are now summarised in supplemental table ST1.

**Reviewer 2, comment 7**: The statistical tests applied appear arbitrary: unpaired t-test versus ANOVA with different post-tests. Why is this not consistent throughout the figures?

During analysis, t-tests (paired or unpaired, as appropriate) were routinely utilised for pairwise data comparisons, while ANOVA was applied for carrying out all possible pairwise comparisons across larger datasets where this analysis was deemed appropriate. We now realise that we utilised only pairwise comparisons against a single control value (Water) for Figure 4C in the final version of the manuscript, and hence ANOVA is not necessary for this analysis. We have updated the manuscript to utilise a t-test here instead. Note that the transport data for Ala-Phe was below the threshold of significance in the previous analysis, but is found to be significant (p<0.05) via the unpaired t-test utilised here, and the figure has been updated to reflect this.

**Part III – Minor Issues: Editorial and Data Presentation Modifications**

R1: “What might be the biology behind the data that DtpT transports GSH but not GSSH? Do other glutathione transporters function in this manner.”

Based on our existing functional understanding of POT transporters, we would not predict that GSSG is a valid substrate for a transporter of this family primarily due to substrate size constraints (this is already mentioned in the manuscript, line 310).

That being said, the existence of redundant routes of GSH uptake (but not GSSG) may suggest that this form of the metabolite is a more valuable nutrient source in relevant growth environments. Recent work has similarly demonstrated that Opp - a promiscuous peptide transporter of *E. coli* - can also transport GSH but not GSSG. Hence, the observation of redundant transporters for GSH in a major bacterial pathogen is not unique. We have added a few lines addressing this point in the discussion section (line 546).

Source; Knoke et al. (2024) 10.1016/j.redox.2024.103453.

R2: “Typos and style issues-

In the author summary: “fulfill”, “tree of life”

“Are widely distributed in staphylococci.”

In the sentence “To compare DtpT-mediated transport of peptides directly, we expressed and purified S. aureus DtpT recombinantly from E. coli cells (Figure S2) and...” I recommend to spell out the species name of E. coli since it was used here first in the manuscript.”

These changes have been implemented in the manuscript

Figure 1:
1D) The authors should state what a “signal curves” exactly is. What was measured here? The use of means and “error bars” is misleading as these are not biological replicates but rather individual samples so other statistics might be more appropriate here? Again it is not clear how often the experiment was repeated.

The definition of a signal curve has been clarified in Figure 1, and the use of the phrase “error bars” has been omitted (now listed as simply “± standard deviation”)

Figure 2:
The authors use 12 + colours in their figure. I suggest to reduce the number of colours for example by using other means such as shadings or patterns. Please further clarify the columns and rows of the matrix (N-term and C-terminus)

Additional labels have been implemented to figure 2 in order to improve readability.

Figure 4:
Figure 4A. This figure needs a clearer legend. I suggest to specify the icons (star, double-circle etc.). Figure B and C. The color shading of the datapoints indicate the results of an ANOVA test. However there is no reference or legend to which colour is significant. Figure B why are the standard deviations omitted from the lines? In figure 4C there seems to be lack of a positive control. Is there a known and confirmed dipeptide which shows strong uptake by DtpT which could be utilized for this purpose?

The legend for Figure 4 has been expanded to implement the reviewer’s recommendations. Significance is now denoted by standard * */* ** / *** annotation.

The inclusion of a strict “positive control” is not possible for a system wherein transport substrates have never been demonstrated biochemically. Here, we instead included three peptides for which DtpT-specific utilisation was previously demonstrated in growth assays (arg-phe/arg-ala/arg-asp) to serve as validation of the methodology.

Figure 6:
What do the shown boxes and whiskers indicate? This is not indicated in the figure legend.

The legend has been updated to include this detail.
